# Supplementary material for: A Method for Mitigating Degradation Effects on Polyamide Textile Yarn During Mechanical Recycling
Source: Polymers (Basel). 2025 Dec 5;17(24):3243. doi: 10.3390/polym17243243 (PMC12736912; doi:10.3390/polym17243243)
Supplement: Supplementary file 1 [file polymers-17-03243-s001.zip › polymers-3999204-supplementary.pdf]

Supplementary Material

A Method for Mitigating Degradation Effects on Polyamide Textile Yarn During Mechanical Recycling

Petra Drohsler [1], Martina Pummerova [1,*], Dominika Hanusova [1], Daniel Sanetrnik [1], Dagmar Foldynova [1], Jan Marek [2], Lenka Martinkova [2] and Vladimir Sedlarik [1,*]

[1] Centre of Polymer Systems, University Institute, Tomas Bata University in Zlín, Trida Tomase Bati 5678,
760 01 Zlín, Czech Republic; drohsler@utb.cz (P.D.); d_hanusova@utb.cz (D.H.); dsanetrnik@utb.cz (D.S.); d_sasinkova@utb.cz (D.F.)

[2] INOTEX, spol. s r.o., Stefanikova 1208, 544 01 Dvur Kralove nad Labem, Czech Republic;
marek@inotex.cz (J.M.); martinkova@inotex.cz (L.M.)

* Correspondence: pummerova@utb.cz (M.P.); sedlarik@utb.cz (V.S.)

Table S1. List of major target compounds identified with high confidence (>85%) by scanning NIST11 Spectra Library, as discerned under an inert (He) atmosphere and sorted by their RT.

No.	Rt./min	Compound	Structure
1	6.99	Cyclopentanone	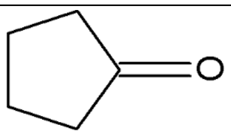
2	15.53	1,8-diazacyclotetradecane-2,7-dione	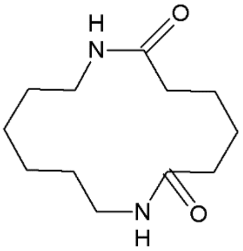

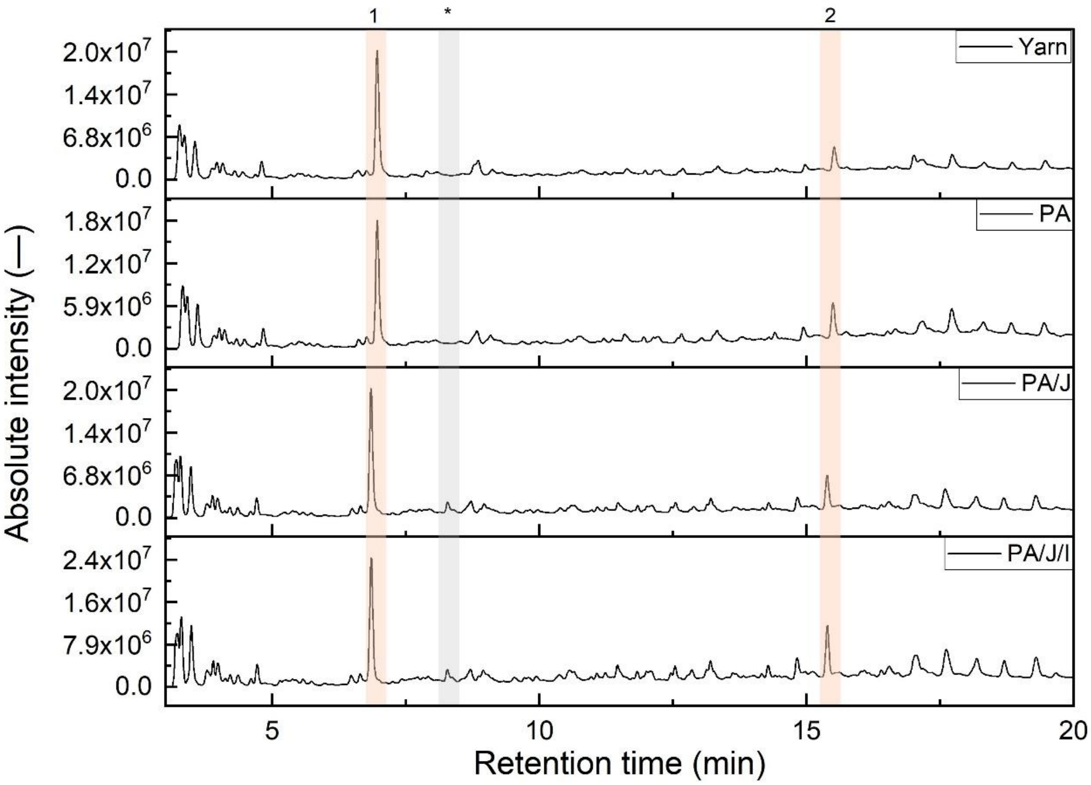

Figure S1. Chromatograms of the samples (after the 1st recycling cycle) recorded under an inert (He) atmosphere by GC/MS.

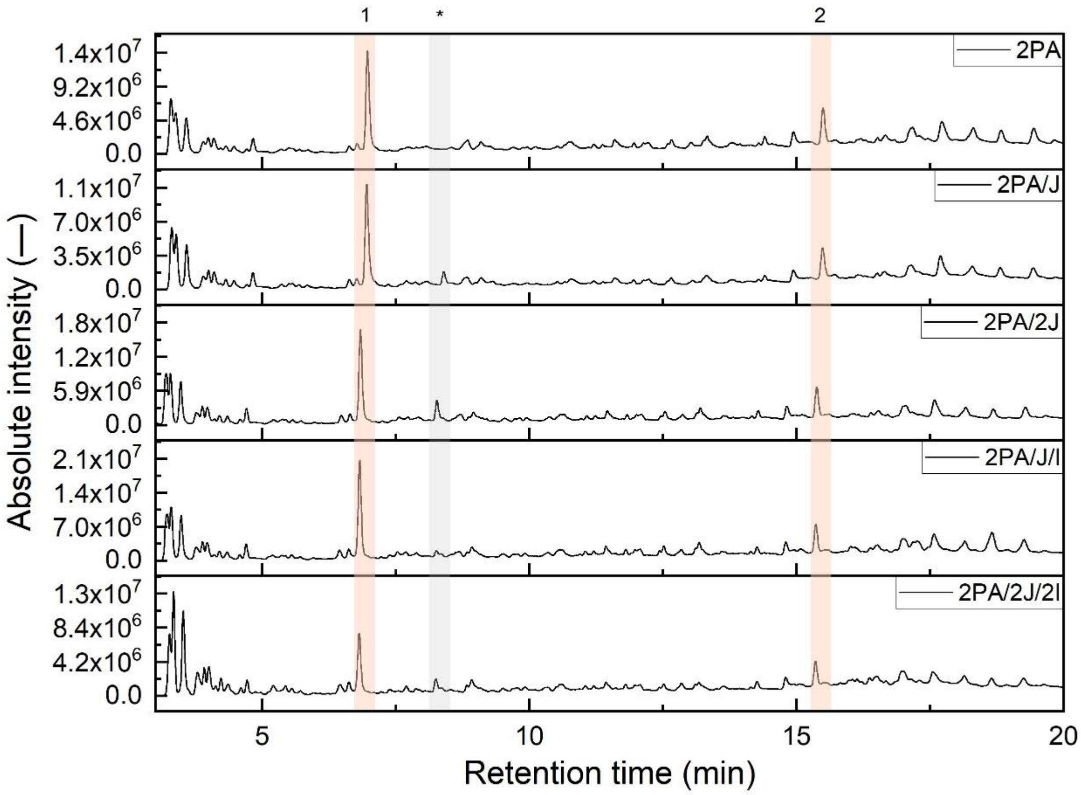

Figure S2. Chromatograms of the samples (after the 2nd recycling cycle) recorded under an inert (He) atmosphere by GC/MS.
